# Efficiency of potato genome editing: Targeted mutation on the genes involved in starch biosynthesis using the CRISPR/dMac3-Cas9 system

**DOI:** 10.5511/plantbiotechnology.23.0611a

**Published:** 2023-09-25

**Authors:** Hiroaki Kusano, Ami Takeuchi, Hiroaki Shimada

**Affiliations:** 1 Laboratory of Plant Gene Expression, Research Institute for Sustainable Humanosphere, Kyoto University, Uji, Kyoto 611-0011, Japan; 2 Graduate School of Frontier Sciences, The University of Tokyo, Kashiwa, Chiba 277-8562, Japan; 3 Department of Biological Science and Technology, Tokyo University of Science,Tokyo 125-8585, Japan

**Keywords:** CRISPR/dMac3-Cas9, genome editing, multiple gRNAs, potato, tuber starch

## Abstract

Potato (*Solanum tuberosum* L.) has a tetraploid genome. To make a mutant lacking a specific gene function, it is necessary to introduce mutations into all four gene alleles. To achieve this goal, we developed a powerful genome editing tool, CRISPR/dMac3-Cas9, which installed the translation enhancer dMac3 that greatly increased the translation of the downstream open reading frame. The CRISPR/dMac3-Cas9 system employing three guide RNAs (gRNAs) greatly elevated the frequency of the generation rate of mutation. This system enabled to create the 4-allele mutants of granule-bound starch synthase (GBSS) and starch branching enzyme (SBE). These mutants indicated functionally defective features, suggesting that we succeeded in efficient genome editing of the potato tetraploid genome. Here, we show the effect of the number of gRNAs for efficient mutagenesis of the target gene using the mutants of the *GBSS1* gene. CRISPR/dMac3-Cas9 employing three gRNA genes achieved a higher mutation efficiency than the CRISPR/dMac3-Cas9 with two gRNAs, suggesting being influenced by the dose effect of the number of gRNAs at the target region. The alleles of the *SBE3* gene contained SNPs that caused sequence differences in the gRNAs but these gRNAs functioned efficiently. However, many rearrangement events and large deletions were induced. These results support the importance of accurate binding of gRNA to the target sequence, which may lead to a hint to avoid the unexpected mutation on the off-target sites.

## Introduction

Potato (*Solanum tuberosum* L.), one of the most important crops, propagates mainly by vegetative reproduction via tubers. Many cultivars are autotetraploid and common potato cultivars have 4n=48 chromosomes. To establish the mutant phenotype, four genome editing events should occur in the homologous chromosomes. In addition to a limited genomic information, genetic diversity is obviously large among major cultivars. It is necessary to develop transformation methods for each potato cultivar to establish a genetically engineered plant (Ohnuma et al. 2020). Therefore, it is difficult to establish a deficient potato mutant. Among common cultivars, the potato cultivar ‘Sayaka’, a model to develop the method, is relatively easy to regenerate, and there have been many reports of the creation of genome-edited potato mutants (e.g., Takeuchi et al. 2021).

The first successful genome editing in potato was reported in 2014 (Sawai et al. 2014). To date, there are many studies on potato genome editing, involving functional analysis of specific genes, the crop breeding toward alterations of desired traits and improvement in the targeted mutagenesis. Reports on the potato genome editing are summarized in Supplementary Table S1.

Potato tuber accumulates a large amount of starch. Storage starch in plants consists of amylose and amylopectin, the composition ratio of which alters starch properties. Granule-bound starch synthase (GBSS) is a major enzyme involved in amylose biosynthesis (Nelson and Rines 1962). Mutants lacking this enzyme produce amylose-free starch. Mutants lacking the *GBSS* gene are known in many plant species, such as rice, maize, and barley (Rohde et al. 1988; Sano 1984; Shure et al. 1983). To date, a knockdown transformant was created by introduction of the antisense gene of *GBSS1* to create amylose-free potato starch (Visser et al. 1991).

Amylopectin is synthesized in concert by the activities of soluble starch synthase (SS) and starch branching enzyme (BE) (Guan et al. 1995). Many kinds of starch branching enzymes have been reported in potato (Larsson et al. 1996; Van Harsselaar et al. 2017). SBE2 and SBE3 are the counterparts of rice BEIIb and BEI. A transformant harboring an antisense gene to *SBE2* exhibits an amylose extension trait (Jobling et al. 1999; Tuncel et al. 2019). Potato SBE3 shows high similarity to rice BEI and is considered the ortholog of rice BEI, which works as a major starch branching enzyme gene (Takeuchi et al. 2021; Van Harsselaar et al. 2017).

To create a deficient mutant of a desired potato gene, a powerful genome editing tool is needed. We have found a translational enhancer, dMac3, that is derived from part of the 5′-untranslated region (5′UTR) of the rice *OsMac3* gene and that strongly elevates the translation efficiency of the downstream ORF (Aoki et al. 2014). Using this approach, an improved CRISPR/Cas9 system has been established and applied to create a potato mutant lacking GBSS activity (Kusano et al. 2018).

We have established the CRISPR/dMac3-Cas9 system employing multiple gRNAs that were targeted to the gene involved in starch biosynthesis. Using them, we evaluated the genome editing efficiency and specificity of the mutants of the *GBSS1* and *SBE3* genes. We determined the influence of the one-nucleotide difference in the gRNA that occurred on an allele of the target gene. In this review, we discuss the optimized methods for genome editing in potato. This paper shows the improved procedure to create a genome-edited potato mutant in which mutations occur in all four alleles of the desired gene.

## Construction of the CRISPR/dMac3-Cas9 containing multiple gRNA genes that are friendly to the cleaved amplified polymorphic sequence (CAPS) analysis

The nucleotide sequence of the potato genome has been first reported in a diploid cultivar of *S. phureja* (http://spuddb.uga.edu). There is a large divergence on the nucleotide sequences among the alleles of the individual genes; a large number of SNPs exists in the alleles in the tetraploid genome. These SNPs enable us to know differences in the alleles of the target gene that will be genome edited.

The potato cultivar Sayaka is commonly used for genome editing. Because Sayaka has a tetraploid genome, genome-editing events may occur on each of four alleles. Detection of mutant alleles in transformants can be achieved using the conventional CAPS analysis. The frequency of generated mutations was determined using a restriction endonuclease-digested/undigested PCR-amplified fragment containing the target sites, as there was a recognition site of the appropriate restriction nuclease in the region corresponding to the gRNA target sequence.

**Figure 1:**
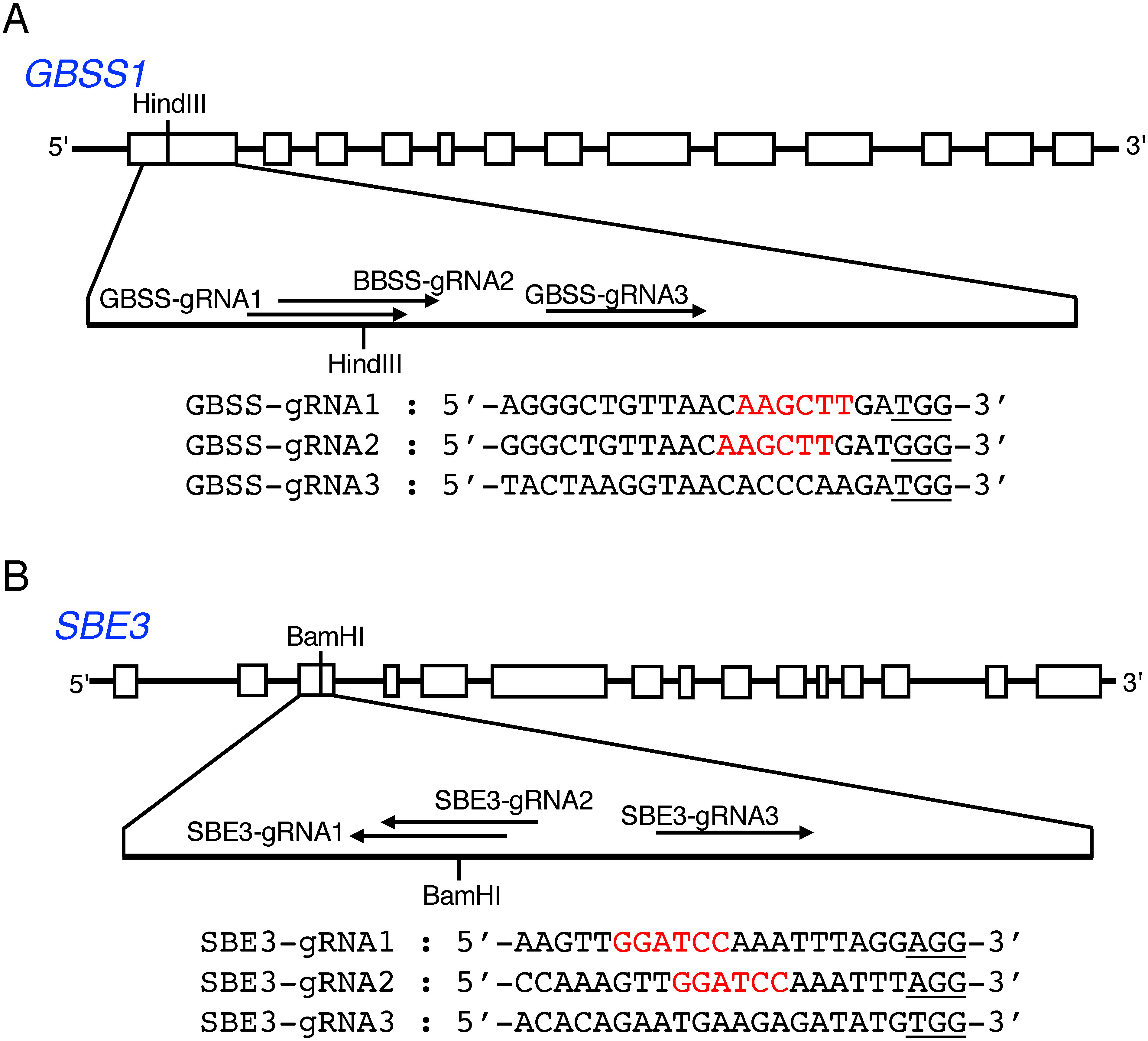
Figure 1. Structure of the *GBSS1* and *SBE3* genes and gRNAs for genome editing. Panels A and B represent *GBSS1* and *SBE3*, respectively. The upper layer in each panel shows a schematic representation of the gene structure. Boxes indicate the exons. HindIII and BamHI sites used for the CAPS analysis are indicated. The lower layer in each panel shows the nucleotide sequence of the target region, on which gRNAs are indicated by arrows. Predicted PAM sequences are underlined.

**Figure 2:**
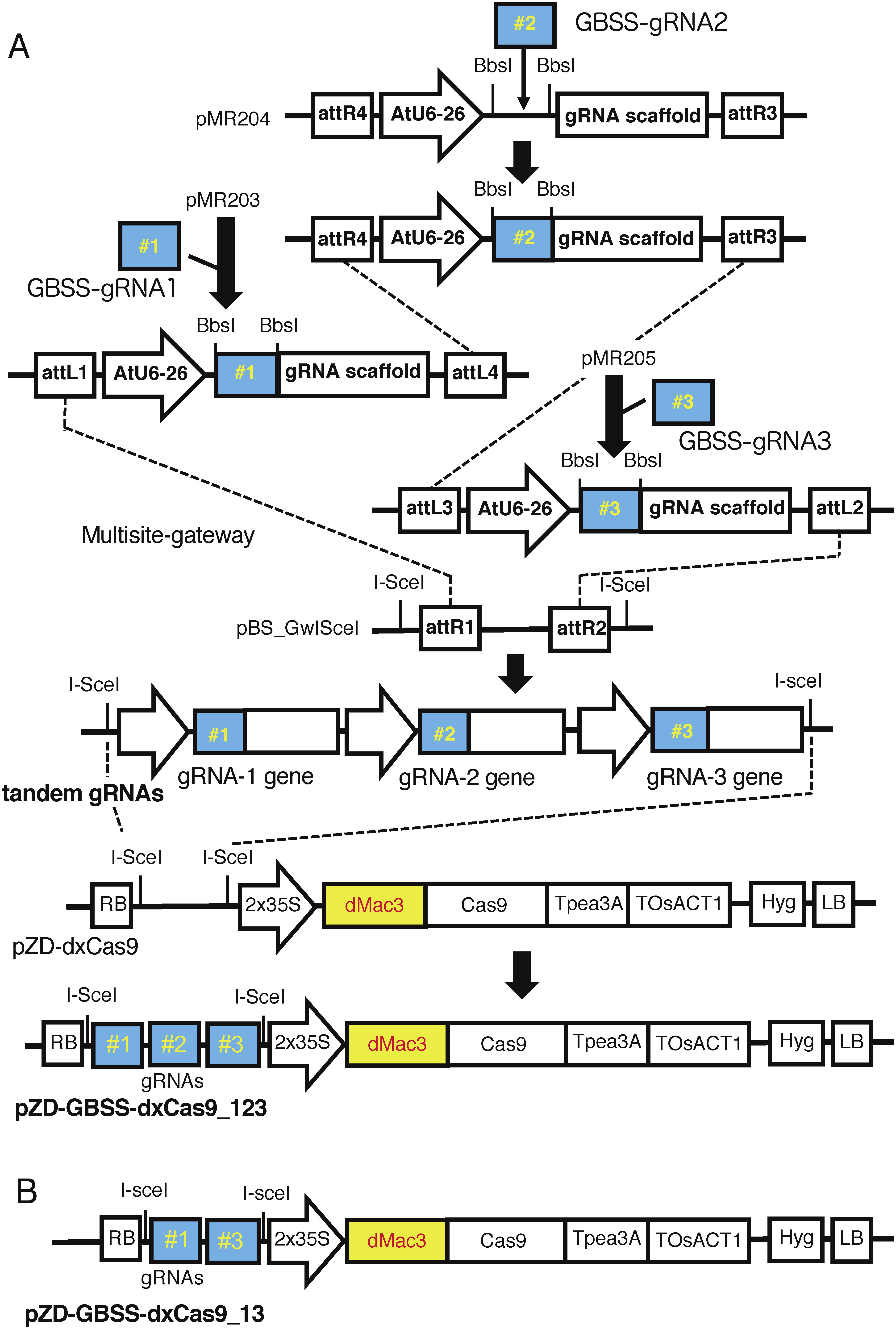
Figure 2. (A) Schematic representation of the construction of the artificial nuclease gene using the CRISPR/dMac3-Cas9 system. The case of construction of an artificial nuclease gene with three gRNAs for the *GBSS1* gene is shown. Chemically synthesized DNAs for the gRNAs were inserted into the BbsI site downstream of the AtU6 promoter. The resultant gRNA genes were located in tandem in pBS_GwIsceI, and they were transferred to pZD-dxCas9 to create a CRISPR/dMac3-Cas9 plasmid, pZD-GBSS-dxCas9_123. (B) Structure of a CRISPR/Cas9 plasmid pZD-GBSS-dxCas9_13, which was constructed by introducing gRNA1 and gRNA3 using multi-gateway method without the integration operation of gRNA2 and otherwise by the similar way.

In the *GBSS1* gene that was involved in amylose synthesis in tuber starch, there was a HindIII site in the first exon. Based on the registered nucleotide sequence of the *GBSS1* gene (accession number (acc. no.) NW_006238976), the gRNA candidates GBSS-gRNA1, GBSS-gRNA2 and GBSS-gRNA3 were chosen as the target sites lying around the HindIII site (Kusano et al. 2018). Among these gRNAs, GBSS-gRNA1 and GBSS-gRNA2 were designed to overlap with one nucleotide slip (Figure 1A). The DNA fragments corresponding to these gRNAs were introduced between the AtU6-26 promoter and the chimeric single-guide RNA (gRNA) scaffold (Li et al. 2013; Mali et al. 2013). Then, two or three of the resultant gRNA genes were introduced into pZD-dxCas9, and the plasmid for the artificial nuclease gene tandemly employing two or three gRNA genes, named pZD-GBSS-dxCas9_13 containing GBSS-gRNA1 and GBSS-gRNA3 or pZD-GBSS-dxCas9_123 containing all gRNAs, was created (Figure 2).

For targeted mutagenesis of the *SBE3* gene of *S. tuberosum*, three regions, SBE3-gRNA1, SBE3-gRNA2 and SBE3-gRNA3 that corresponded to the sequences within the third exon of the *SBE3* gene were selected (Takeuchi et al. 2021). SBE3-gRNA1 and SBE3-gRNA2 were designed to overlap at the position shifted by three nucleotides. In the region of the target sequences, there was a BamHI site, which facilitated the detection of targeted mutations by CAPS analysis (Figure 1B). The sets of the fragments of these gRNAs were introduced downstream of the AtU6-26 promoter, and the resultant gRNA genes were introduced into the Cas9 vector pZD-dxCas9, resulting in the formation of the CRISPR/dMac3-Cas9 vector pZD-SBE3-dxCas9 containing three gRNA genes.

## Effect of multiple guide RNA genes to the efficiency of genome-editing

The created artificial nuclease gene was introduced into the stems of potato plants. Among the regenerated potato transformants, mutants of the *GBSS1* gene were selected. There was a HindIII site in or near the target sites, and therefore, the occurrence of targeted mutations was screened by detecting the disruption of this HindIII site. The DNA fragment containing the target site was PCR-amplified, and CAPS analysis was carried out by HindIII cleavage. We obtained 45 lines of potato mutants in total from the 57 regenerated plants. Among them, 10 lines were suggested to be mutants in which all of four alleles of the *GBSS1* gene were disrupted (Table 1).

**Table 1:** Table 1. Estimated allele numbers of mutants in the *GBSS1* gene in the transformants.

Number of mutant alleles	Number of gRNAs			
	With dMac3		Without dMac3	
	Two	Three	Two	Three
0	6	6	7	6
1	7	5	3	3
2	8	4	0	5
3	5	6	0	6
4	2	8	0	1
Total	28	29	10	21

The number of mutant alleles was estimated by the CAPS analysis of the mutant lines. Mutant lines detected by the genome editing using two and three gRNAs in the CRISPR/dMac3-Cas9 (left) and conventional CRISPR/Cas9 (right) vectors are shown.

We compared the ratio of the targeted mutagenesis by the CRISPR/Cas9 vectors containing two or three guide RNA genes and the effects of the gRNA gene dosage introduced for the generation of *GBSS1* gene mutations. The CRISPR/dMac3-Cas9 with three gRNAs showed higher efficiency for targeted mutagenesis than those with two gRNAs (Table 1). In this case, 23 of 29 (75%) transformants had mutant *GBSS1* genes, with 8 transformants (28%) showing the mutation at all four alleles. However, the ratio of generation of four allele mutants was 7% (2 of 28) when using two gRNAs, although the ratio of mutant generation was 79% (22 of 28), which was similar to the ratio of mutant generation with three gRNAs (Table 1). These results demonstrated that the CRISPR/dMac3-Cas9 vector containing three gRNAs was more efficient in targeted mutagenesis at the multiple alleles than the vector containing two gRNAs.

Similarly, genome-edited mutants of the *SBE3* gene were generated using pZD-SBE3-dxCas9. Because there was a BamHI site in the region of the gRNAs, the occurrence of targeted mutations was detected by disruption of this BamHI site. The CAPS analysis was carried out by BamHI cleavage of the PCR-amplified fragment containing the target site. We obtained 88 lines of potato mutants in total. The ratio of mutant generation was 70% in 125 regenerated lines. Among them, 10 lines were estimated as the four allele mutants of the *SBE3* gene (Table 2).

**Table 2:** Table 2. Estimated allele numbers of mutants in the *SBE3* gene in the transformants.

Number of mutant alleles	Number of mutants
0	37
1	25
2	34
3	19
4	10
Total	125

The number of mutant alleles was estimated by the CAPS analysis of the mutant lines. Mutant lines detected by the genome editing are shown.

## Sequence analysis of the target sites in the mutant alleles of the *GBSS1* gene

**Figure 3:**
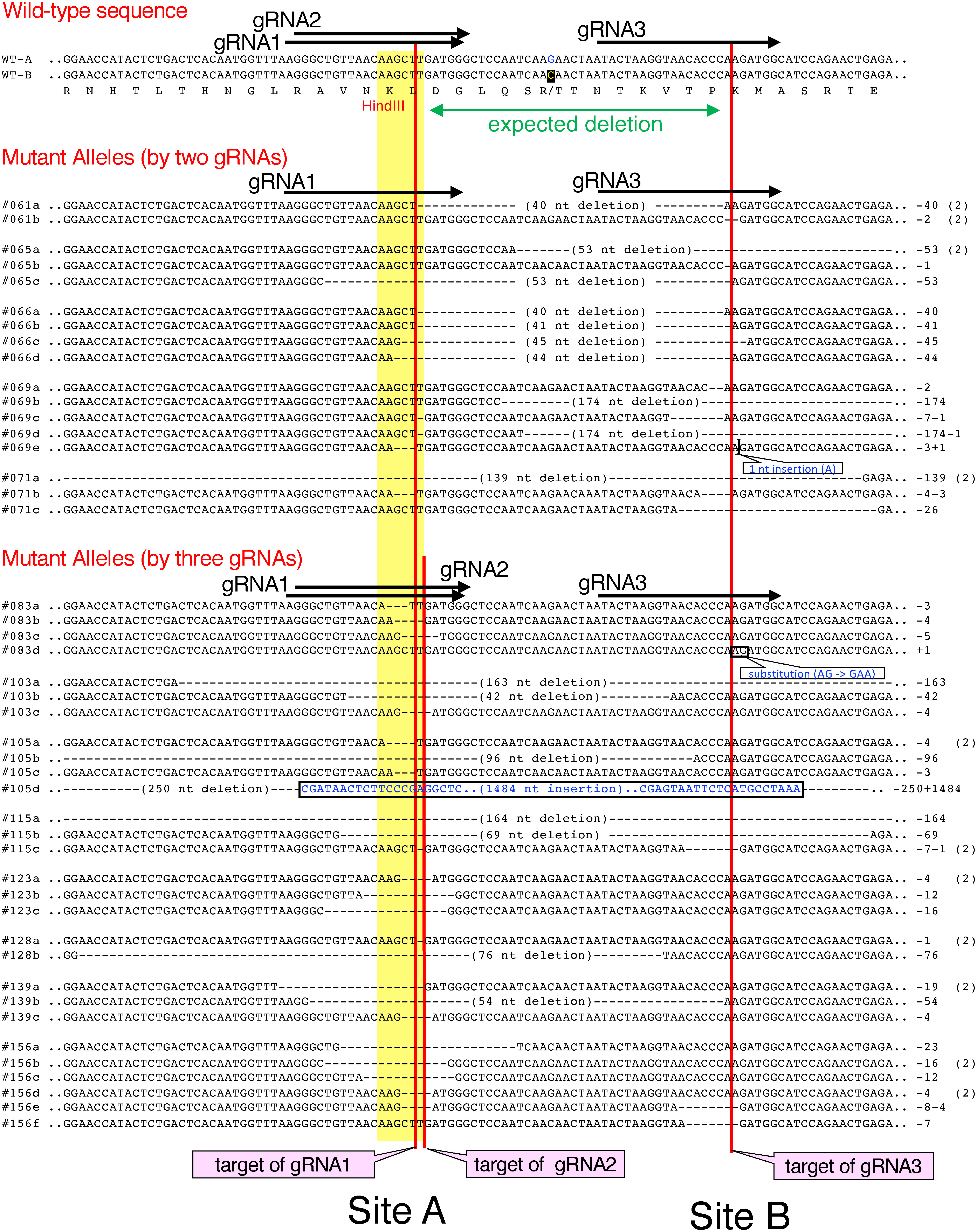
Figure 3. Nucleotide sequences of the mutant alleles of the *GBSS1* gene generated by genome editing using two or three gRNAs. Nucleotide sequences near the target sites of the representative mutants are shown. The nucleotide showing the SNP in WT-B to that in WT-A is indicated in yellow letters in black. Numbers prefixed with # indicate the names of mutant lines, and multiple mutant alleles are distinguished by the addition of “a” to “g”. The numbers of deleted and inserted nucleotides are shown on the right. Mutants consisting of the same sequences found in different alleles are marked with (2) on the right side. Sites of gRNAs are indicated by arrows. Names of gRNAs in the figure are shown without the prefix “GBSS-”. Red vertical lines indicate the predicted cleavage sites by Cas9. The yellow box indicates the HindIII site used for CAPS.

We determined the nucleotide sequences of the target regions in the *GBSS1* gene from the representative mutants to detect polymorphic DNA sequences around the mutant target regions. As shown in Figure 3, nucleotide deletion or insertion was found at the region of the gRNAs, which included large deletions or insertions in the region between/beyond the positions of gRNAs. A large rearrangement event was found in a mutant allele of #105 (#105d in Figure 3), which contained a 250-nt deletion and a 1,484-nt insertion. The sequence of this insertion showed similarity to unidentified sequence derived from *S. tuberosum* (Supplementary Figure S1).

We investigated the difference in susceptibility to mutation depending on the number of gRNA genes included in the artificial nuclease genes. For this experiment, we used three gRNAs that targeted the region near the HindIII site in the *GBSS1* gene (Figure 1). Among the gRNAs, two gRNAs, GBSS-gRNA1 and gRNA2, were expected to recognize the position near the HindIII site to digest at the +2 and +3 positions from the HindIII site (Site A in Figure 3). Their recognition sites essentially overlapped each other. However, the target of GBSS-gRNA3 was the +44 position from the HindIII site (Site B in Figure 3). We constructed two kinds of artificial nuclease genes: one contained two gRNAs, GBSS-gRNA1 (Site A) and GBSS-gRNA3 (Site B), and the other contained three gRNAs, GBSS-gRNA1 (Site A), GBSS-gRNA2 (Site A) and GBSS-gRNA3 (Site B).

Mutation of the *GBSS1* gene was detected by cleaving the PCR-amplified fragment of the gRNA region with HindIII. Because the HindIII site is located near the region of GBSS-gRNA1 and GBSS-gRNA2, this method might lose the chance to detect any mutation occurring in the GBSS-gRNA3 region. However, it was assumed that this underestimation did not strongly affect to the results. In this case, many mutations detected occurred in both two sites and spanned over them (Figure 3). This result indicated that two or more genome editing events independently occurred in these mutants. This result suggests that each gRNA worked well for the genome editing in this experiment.

**Figure 4:**
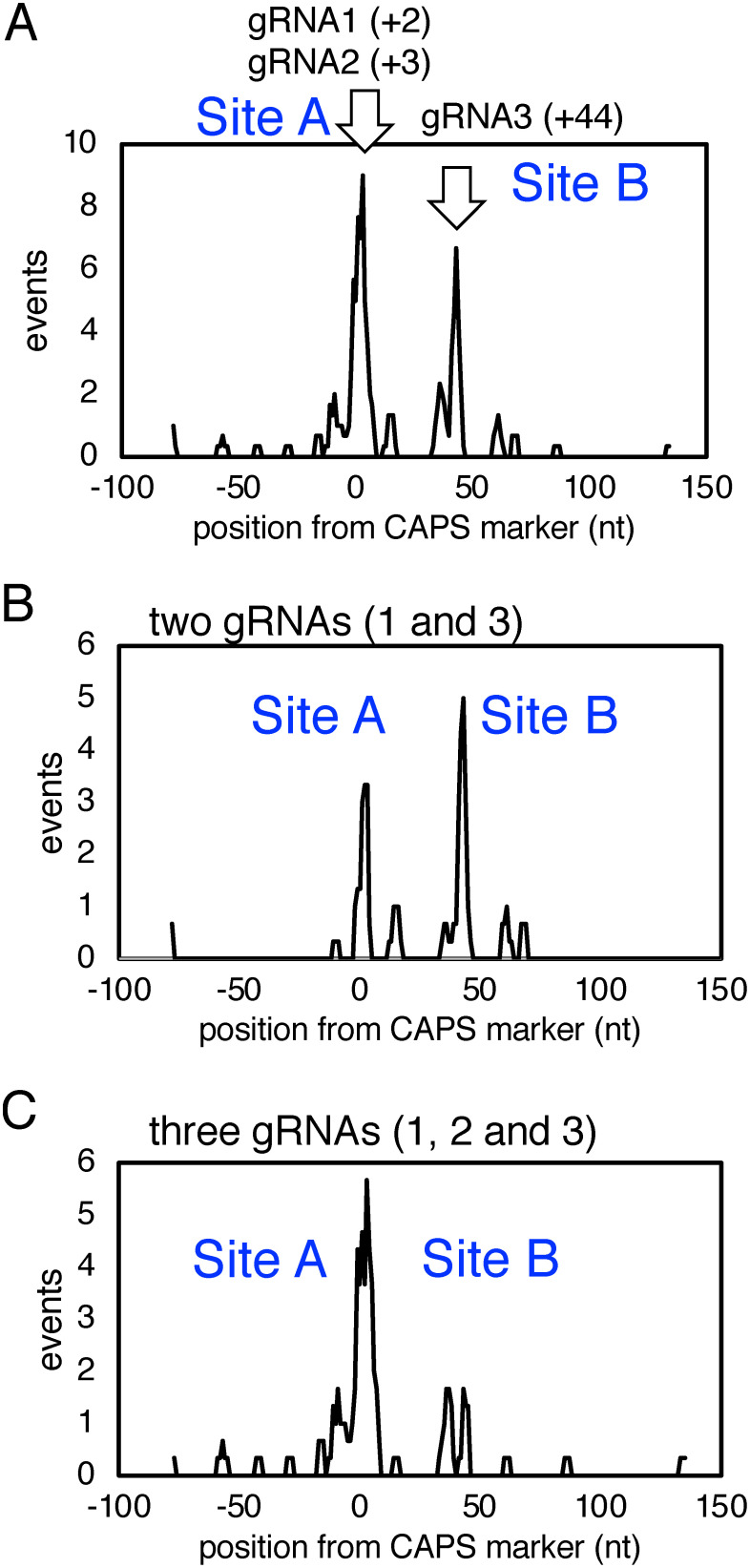
Figure 4. Frequency of genome editing events depending on positions around the target region in the *GBSS1* gene. The horizontal axis indicates the position from the 0 position in the HindIII site. The vertical axis indicates the frequency of genome editing events at each position. The number of events represents the average value that was convoluted at 3-base resolution in the contiguous ranges. Site A corresponds to the target sites of gRNA1 and gRNA2 (+2 and +3), and Site B is the target site of gRNA3 (+44). (A) Frequency of genome editing events depending on the position in the *GBSS1* genes. All mutation events caused by genome editing using two or three gRNAs are summarized. (B) Frequency of the site-dependent genome-editing events when using two gRNAs. (C) Frequency of site-dependent genome-editing events when using three gRNAs.

Numerous deletions occurred in the regions corresponding to the target sites, Site A and Site B (Figure 4). When two gRNAs were used, 29% and 42% of genome editing events were detected at Site A and Site B, respectively (Figure 4B). When genome editing was performed using three gRNAs, 53% and 17% of the genome editing events occurred at Site A and Site B, respectively (Figure 4C). These results indicated that genome-editing events more frequently occurred at Site A when using GBSS-gRNA1 and GBSS-gRNA2, which are located in the overlapping region of Site A. A mutation lacking the region between two target sites, Site A and Site B, was found in many mutant genes. This 40- or 41-nt deletion was an expected mutation that could be generated when cleavages at two sites occurred simultaneously. Such mutant genes appeared frequently when two gRNAs were used for genome editing (Figure 4B). In addition, mutated genes lacking very large regions were also observed frequently (Figure 4C).

## Sequence analysis of the target sites in the mutant alleles of the *SBE3* gene

**Figure 5:**
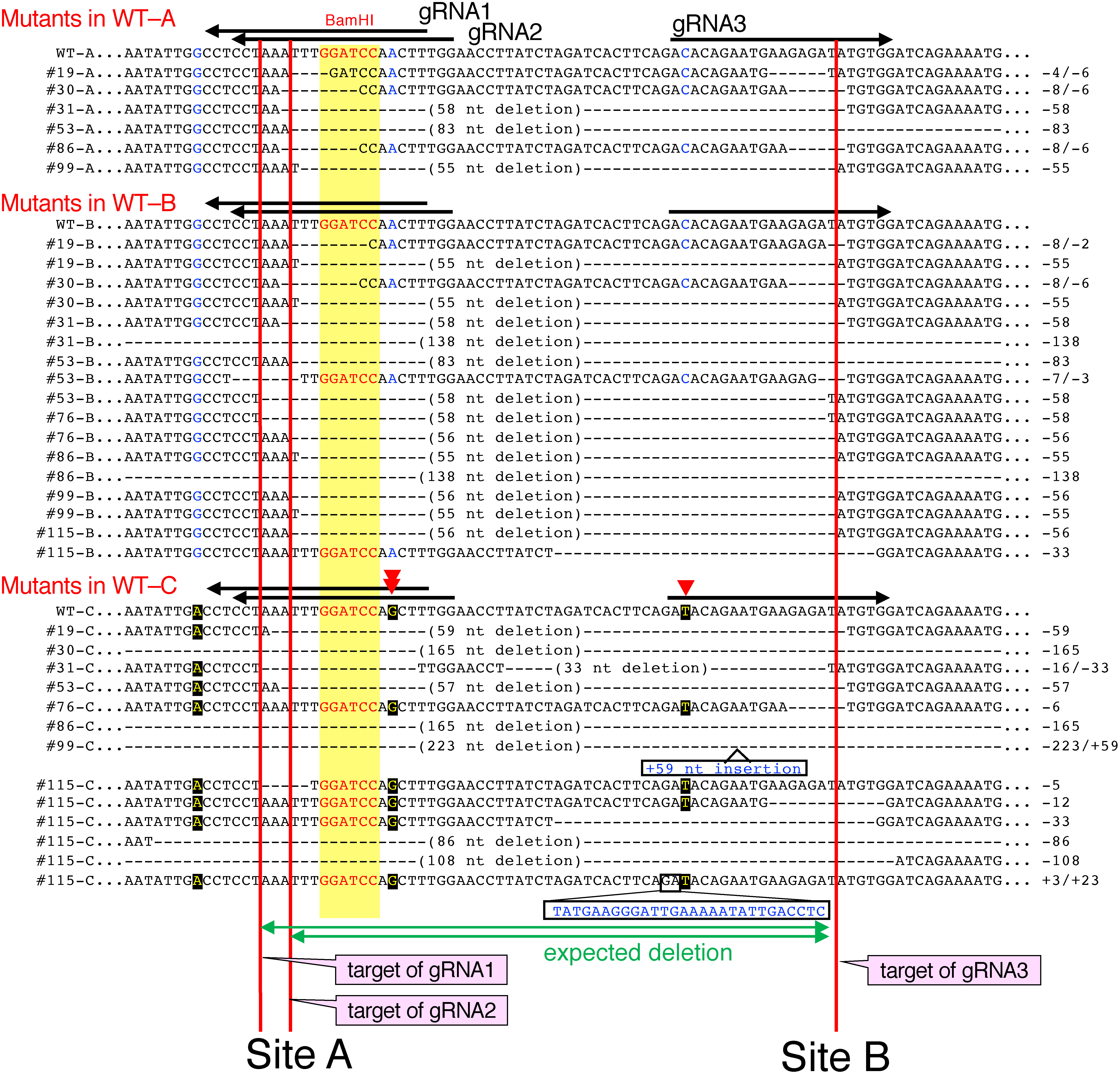
Figure 5. Nucleotide sequences of the mutant alleles detected in WT-A, WT-B and WT-C of the *SBE3* gene. Nucleotides in WT-C that show SNPs to those of WT-A and WT-B are indicated in yellow letters in black. Nucleotide sequences near the target sites of the representative mutants are shown. Numbers prefixed with # indicate the names of mutant lines. The numbers of deleted and inserted nucleotides are shown on the right. Sites of gRNAs are indicated by arrows. Names of gRNAs in the figure are shown without the prefix “SBE3-”. Red triangles indicate the points of the single nucleotide difference in the gRNAs to the WT-C sequence. Red vertical lines indicate the predicted cleavage sites by Cas9. The yellow box indicates the BamHI site used for CAPS.

The *SBE3* gene of Sayaka showed a polymorphism at the sequences in the target site region, suggesting that there were three different nucleotide sequences, named WT-A, WT-B, and WT-C (Figure 5). They were presumed to exist in a 1 : 2 : 1 proportion in the four alleles (Takeuchi et al. 2021). Among these alleles, the nucleotide sequences of WT-A and WT-B were identical in the region around the target region, but a single nucleotide difference was present in each of the gRNAs compared to WT-C (Figure 5).

Similar to genome editing on the *GBSS1* gene, we set two gRNAs, SBE3-gRNA1 and SBE3-gRNA2, at the -6 and -9 positions that overlapped near the BamHI site (Site A in Figure 5). SBE3-gRNA3 was located 50 nt from the BamHI site (Site B in Figure 5). The nucleotide sequences of the representative *SBE3* mutants indicated that mutation events occurred mainly in the region near the sites of gRNAs. Deletion of the genome sequences was found at the region inside/between the sites of gRNA1, gRNA2 and gRNA3 (Figure 5).

Genome editing events evenly occurred on three genome alleles (Table 3). This result suggested that each gRNA worked equally to induce mutation at the target regions. In WT-A and WT-B, deletions of 55–58 nucleotides covering the regions of Site A and Site B were often found. However, deletions of the large region were detected on WT-C at a higher frequency than the deletion of WT-A and WT-B (Figure 5). In addition, events of insertion were found only in the mutation on WT-C among three types of the genome alleles (Figure 5).

**Table 3:** Table 3. Numbers of mutants in each genome of the *SBE3* gene.

Position of mutation	Genome allele		
	WT-A	WT-B	WT-C
Site A	9	16 (8)	5
Site B	7	17 (8.5)	8
Others	2	7 (3.5)	13
Total	18	40 (20)	26

The number of mutations found at each site was counted. The position of mutation was determined by the nucleotide sequences of each mutant. Numbers in parentheses indicate those of an estimated single allele of WT-B because of two alleles in the genome.

## Perspectives toward the efficient genome editing

Many applications have been shown for CRISPR/Cas9-mediated genome engineering in various organisms. Mutation frequencies differ depending on the *Cas9* expression cassette, and the best combination of *Cas9*/gRNA expression cassette results in an improved frequency of targeted mutagenesis (Mikami et al. 2015). In addition to the high-level expression of the *Cas9* gene, its efficient translation may result in increased efficiency toward targeted mutagenesis due to the generation of large amounts of Cas9 protein. We have reported that the 5′UTR of *OsMac3* (dMac3) acts as a translation enhancer, largely increasing the production of the protein encoded by the downstream open reading frame, leading to a 10-fold or greater increase in its translational efficiency (Aoki et al. 2014; Teramura et al. 2012). The CRISPR/dMac3-Cas9 system uses a portioned fragment of the 5′UTR of *OsMac3* as the translational enhancer for the *Cas9* gene to generate potato mutants with remarkable efficiency (Kusano et al. 2018).

*GBSS1* is typically a model target for genetic engineering in potato. A *GBSS1*-deficient mutant shows amylose-free starch in potato tubers, whose phenotype is easily detected by the iodide staining method (Morrison and Laignelet 1983). A *GBSS1* mutants have been obtained by random mutagenesis (Hovenkamp-Hermelink et al. 1987) and created using antisense genes, RNA interference, transcription activator-like effector nucleases (TALENs), and the CRISPR/Cas9 system (Andersson et al. 2017; Brummell et al. 2015; Kusano et al. 2016; Visser et al. 1991). Potato SBE3 is the counterpart of rice BEI, which is a key enzyme for the production of amylopectin. The mutant lacking BEI showed no significant change in the apparent amylose content, but the structure of amylopectin was largely altered because of a decrease in long chains and an increase in short chains of amylopectin. In addition, the endosperm starch of this mutant displayed altered properties of gelatinization (Satoh et al. 2003).

The efficiency and specificity of targeted mutagenesis was examined on the potato *GBSS1* and *SBE3* genes created by disruption of these genes. They contained various types of polymorphic DNA sequences that contained nucleotide deletions or rearrangements in the *GBSS1* and *SBE3* genes. It has been shown that mutations in the *GBSS1* gene and the *SBE3* gene resulted in a reduction in the amylose content in potato tuber storage starch (Supplementary Figure S2) (Kusano et al. 2018; Takeuchi et al. 2021).

We evaluated the effect of the number of gRNAs for efficient mutagenesis of the target gene using the *GBSS1* mutants. Simultaneous use of two or more gRNAs has been reported to induces the deletion of the region between two gRNAs (Čermák et al. 2017). Our results indicated that CRISPR/dMac3-Cas9 employing three gRNA genes resulted in 4-allelic mutations with higher efficiency than the CRISPR/dMac3-Cas9 with two gRNAs (Table 1). Among the three gRNAs, two gRNAs were located in the overlapping region, and a high frequency of mutation events was found in this region (Figures 3, 4). This suggests being influenced by the dose effect of the number of gRNAs at the target region. Therefore, a high efficiency of genome editing is expected to be achieved when multiple gRNAs are used for the CRISPR/dMac3-Cas9 vector.

The expected genome editing event in the *GBSS1* gene, which induced a deletion of the region between Site A and Site B, was frequently observed in the mutants generated using the artificial nuclease gene with two gRNAs, whereas genome editing using three gRNAs often induced the larger nucleotide deletion in the target region (Figure 3). This observation suggests that CRISPR/dMac3-Cas9 employing two gRNAs will achieve the expected genome editing with higher accuracy than with three gRNAs, although the efficiency of genome editing of 4-allelic mutations is lower than that using three gRNAs (Figure 3).

Nucleotide insertion was also observed in the target region. A mutant allele of *GBSS1* mutant #105d contained a 1.5-kb insertion along with a 250-nt deletion (Figure 3, Supplementary Figure S2). In this case, the inserted sequence was suggested to be derived from the potato genome. This matter suggests that such a genome rearrangement event may often occur during the genome editing process.

The nucleotide sequences of the genes of *S. tuberosum* cv. Sayaka were not registered in the database when the study began. The gRNA genes were constructed based on the registered nucleotide sequences of *S. phureja*. Because the structures of the genes in potato cultivars are largely divergent, the corresponding regions in the Sayaka genome were determined to identify the gRNA sequences. Two different nucleotide sequences WT-A and WT-B were found in the *GBSS1* gene. However, the nucleotide sequences of three gRNAs were matched with the target region, and there were no mismatches between them. The *SBE3* gene consisted of three different nucleotide sequences, WT-A, WT-B, and WT-C due to the existence of SNPs (Figure 5). Among these sequences, WT-C contained SNPs that caused sequence differences in the gRNAs (Figure 5). These gRNAs functioned efficiently even on WT-C (Table 3). Many rearrangement events and large deletions were found in WT-C containing mismatches in the binding site of gRNA to the target sequence (Figure 5). This may give a hint to avoid the unexpected mutation on the off-target sites.

Genome editing is a newly developed technology using a sequence-specific artificial nuclease to enable modification of a specific gene (Osakabe and Osakabe 2015). Whereas the conventional breeding method takes a long time to obtain a variety possessing a desired trait, genome editing technology may facilitate the rapid establishment of an appropriate mutant with a desired trait (Xiong et al. 2015). CRISPR/dMac3-Cas9 is a powerful tool to induce a mutation at the target site with a high editing efficiency. This system is expected to facilitate the efficient genome-editing events in potato, which promotes the metabolic engineering of desired properties, such as traits of tuber starch.

## Data Availability

The original contributions presented in the study are included in the article, further inquiries can be directed to the corresponding author. The plasmids for CRISPR/dMac3-Cas9 system are available from RIKEN BRC (https://dna.brc.riken.jp/en/).

## Description of Supplementary Files

The Supplementary Material for this article can be found online.
