# Supplementary material for: Efficiency of potato genome editing: Targeted mutation on the genes involved in starch biosynthesis using the CRISPR/dMac3-Cas9 system
Source: Plant Biotechnol (Tokyo). 2023 Sep 25;40(3):201–9. doi: 10.5511/plantbiotechnology.23.0611a (PMC10901159; doi:10.5511/plantbiotechnology.23.0611a)
Supplement: Supplementary Data [file plantbiotechnology-40-3-23.0611a-s001.pdf]

SUPPLEMENTARY METHODS

Establishment of the germ-free potato plants and culture condition

Germ-free potato plants were established as follows: A tuber of the potato cultivar "Sayaka" was peeled and then cut into 3-5 mm slices. These sections were immersed in 70% ethanol for 1 minute, soaked in 0.4% sodium hypochlorite solution for approximately 15 minutes, and then rinsed well with sterilized water. The sliced sections were placed on an MS plate and cultured in a growth chamber at 23 °C with a light period of 16 hours (200 µmol m^{-2} s^{-1}) and a dark period of 8 hours. They were transplanted every 1-3 weeks. After 6 to 8 weeks, shoots that germinated from the region of the epidermis, were transplanted to an MS plate in a test tube. Generated plants were used as germ-free plants. Potato plants were cultured on the medium containing a Murashige and Skoog (MS) basal salt mixture (Fuji Film Wako, Tokyo, Japan) (Murashige and Skoog, 1962), 3% sucrose, and 0.3% Gelrite (Fuji Film Wako) with the pH adjusted to 6.0 (MS plate) under germ-free conditions.

Agrobacterium-mediated transformation

Potato was transformed using the *Agrobacterium*-mediated procedure using the *A. tumefaciens* EHA105 strain according to Yamada et al. (2004) and Kusano et al. (2018). *A. tumefaciens* was cultured with shaking at 28 °C for 24 hours in 2xYT liquid medium (Bacto trypton 1.6%, Bacto Yeast extract 0.1%, NaCl 0.5%) supplemented with 50 mg/L spectinomycin (Fuji Film Wako). Cells harvested at the late-stationary phase were resuspended in 2xYT liquid medium to adjust the cell concentration to OD600 = 0.1. Then, 2 µL of 10 mg/mL acetosyringone and 1 mL of 3C5ZR medium (Sheerman et al., 1988) were added to 150 µL of the suspended *A. tumefaciens*. The resultant suspension was used for the potato transformation.

Stem internodes were isolated from germ-free potato plants grown in a clean environment for 3 weeks and cut into approximately 0.7-cm pieces. These pieces were infected with *A. tumefaciens* harboring an appropriate plasmid. After treatment with *Agrobacterium* infection, the stem internodes were rinsed with sterilized water, placed onto 3C5ZR plates containing 0.3% Gelrites, and cultured in a growth chamber at 23 °C

under a long-day conditions with a light period of 16 hours (200 μmol m^{-2} s^{-1}) and a dark period of 8 hours for 4-6 days. Then, they were washed with sterile water.

After this treatment, callus induction and plant regeneration were performed by culturing for 2 months on a plate of 3C5ZR medium (Sheerman et al., 1988) containing 0.3% Gelrite supplemented with 5 mg L^{-1} hygromycin B (Fuji Film Wako). Regenerated shoots were transplanted onto MS plates supplemented with 5 mg/L hygromycin B to generate regenerated plants. The regenerated plants were cultured on MS plates under long-day conditions. Introduction of the transgene into the regenerated plants was confirmed by polymerase chain reaction (PCR) using the primers, 5'–GGCGTAAGAATAGAATCTGTTAT–3' and 5'–GACAGCGCTATCAGATTTCCAA–3', which amplified a part of the *Cas9* gene sequence.

Determination of the properties of tuber starch

Starch granules were prepared from potato tubers according to Noda et al. (2004). Sliced sections of potato tuber were dipped into 0.4% potassium iodide-0.12% iodide solution. Photographs were taken after washing with water. The amylose content in the potato tubers was analyzed according to a previous paper (Noda et al., 2004). The data were statistically analyzed using Dunnett's multiple comparison test.

Sectioned tissues of potato tuber (150 mg) were frozen in liquid nitrogen and ground into a fine powder using an SK mill (Tokken Inc., Kashiwa, Japan). Then, 400 μL of the protein extraction buffer (50 mM Tris-HCl, pH 6.8, 8 M urea, 4% sodium dodecyl sulfate (SDS), 20% glycerol, 1 mM dithiothreitol (DTT), 0.01% bromophenol blue) was added to the powdered tuber. The mixture was well combined and centrifuged at 20,000 x g for 15 min at room temperature. The supernatant solution was collected as a crude protein fraction. This fraction (10 μL) was used for 7.5% SDS-polyacrylamide gel electrophoresis and subjected to Western blot analysis using antiserum raised against rice BEI (Kawasaki et al., 1996). SDS-PAGE and Western blot analysis were performed according to Wakasa et al. (2020). For this analysis, a 5,000-fold dilution of the antiserum was used. Protein interaction with the antibody was detected using the anti-rabbit immunoglobulin G (IgG) horseradish peroxidase (HPR)-linked secondary antibody (1:10,000 dilution) (GE Healthcare, Chalfont Saint Giles, UK). Signals were

detected with Pierce™ ECL Plus Western Blotting Substrate (Thermo Fisher Scientific, Waltham, MA, USA).

REFERENCES

Kawasaki, T., Mizuno, K., Shimada, H., Satoh, H., Kishimoto, N., Okumura, S., Ichikawa, N., Baba, T. (1996) Coordinated regulation of the genes participating in starch biosynthesis by the rice Floury-2 locus. *Plant Phsiol.* 110, 89–96

Kusano, H., Ohnuma, M., Mutsuro-Aoki, H., Asahi, T., Ichinosawa, D., Onodera, H., Asano, K., Noda, T., Horie, T., Fukumoto, K., Kihira, M., Teramura, H., Yazaki, K., Umemoto, N., Muranaka, T., Shimada, H. (2018) Establishment of a modified CRISPR/Cas9 system with increased mutagenesis frequency using the translational enhancer dMac3 and multiple guide RNAs in potato. *Sci. Rep.* **8**, 13753

Murashige, T., Skoog, F.A. (1962) A revised medium for rapid growth and bioassays with tobacco tissue culture. *Physiol. Plant.* **15**, 473–497

Noda, T., Tsuda, S., Mori, M., Takigawa, S., M.-Endo, C., Saito, K., Mangalika, W.H.A., Hanaoka, A., Suzuki, Y., Yamauchi, H. (2004) The effect of harvest date on starch properties in various potato cultivars. *Food Chem.,* **86**, 119–125

Sheerman, S., Bevan, M.W. (1988) A rapid transformation method for *Solanum tuberosum* using binary *Agrobacterium tumefaciens* vectors. *Plant Cell Reports* **7**, 13–16

Yamada, T., Tozawa, Y., Hasegawa, H., Terakawa, T., Ohkawa, Y., Wakasa, K. (2004) Use of a feedback-insensitive α subunit of anthranilate synthase as a selectable marker for transformation of rice and potato. *Mol. Breed.* **14**, 363–373

Wakasa, Y., Kasai, A., Yamazaki, M., Tabei, Y., Tsuyama, M., Igarashi, T., Okazaki, T., Yamamoto, K., Fujihara, H., Kanno, A., Noro, O., Harada, T., Akada, S. (2020) Rapid analysis of *GBSS1* and *Vinv* genes expressed in potato tubers using microtubers produced in liquid culture medium. *Plant Cell Rep.* **39**, 1415–1424

Supplementary Table S1. Genome editing studies of genes involved in crop trait of potato

Target gene	Description (product phenotype, gene function, research aim, etc.)	Cultivar/variety/line	Reference
Starch composition change			
GBSSI	amylose-free starch	Yukon Gold	Toinga-Villafuerte et al. 2022 Int J Mol Sci 23: 4640
GBSS	amylose-free starch	Desirée, Wotan	Johansen et al. 2019 Sci Rep. 9: 1771
StGBSSI	amylose-free starch	Desirée, Furia	Veillet et al. 2019 Plant Cell Rep 38: 1065–1080
GBSS	amylose-free starch	Kuras	Andersson et al. 2018 Physiol Plant. 164: 378–384
StGBSS	amylose-free starch	Kuras	Andersson et al. 2017 Plant Cell Rep 36:117–128
GBSSI	amylose-free starch	Sayaka	Kusano et al. 2018 Sci Rep 8: 13753
GBSSI	used as a model target gene to test new technology	Desirée	Veillet et al. 2020 Int J Mol Sci 20: 402
StGBSS	used as a model target gene to test new technology	Desirée	Zong et al. 2018 Nat Biotechnol 36:950–953
GBSS	used as a model target gene to test new technology	Sayaka	Kusano et al. 2016 Sci Rep 6: 30234
SBE3	apparently amylose-rich starch, more sensitive to iodine staining	Sayaka	Takeuchi et al. 2021 Plant Biotechnol 38: 345–353
SBE1, SBE2	amylopectin-free starch in double full-allelic mutation line	Desirée	Zhao et al. 2021 Sci Rep 11: 4311
SBE1, SBE2	range of starch properties, longer chain length, fewer blanch, smaller and many granules	Desirée	Tuncel et al. 2019 Plant Biotechnol J 17: 2259–2271
SS6	unknown isoform of starch synthase, discovered by genome analysis	Desirée	Sevestre et sl. 2020 Sci Rep 10: 2045
GWD1	key regulatory enzyme in starch metabolism, hypothetical	Saturna	Carlsen et al. 2022 Front Genome Ed 3: 795644
Reducing acrylamide-generation, cold-induced sweetwening (CIS) and bud outgrowth			
VInv	reduced CIS, lipid oxidation and H_2O_2 level in tuber; reduced plant wilting in dry environment	Desirée, Brooke	Teper-Bamnolker et al. 2022 Plant J 113: 327–341
VInv	reduced CIS by partial knockdown/silencing	AGB Purple	Yasmeen et al. 2022 Planta 256: 107
StvacINV1, StBAM1	postharvest-related potential target genes	Yagana-INIA	Acha et al. 2021 Plants 10: 1882
VInv	revealing molecular system consist of VInv, cytokinin and sugar, involved in lateral bud growth	Desirée	Salam et al. 2021 Plant Physiol 185: 1708–1721
VInv	reduced acrylamide production upon high-temperature processing	Ranger Russet, Atlantic, Russet Burbank, Shepody	Clasen et al. 2016 Plant Biotechnol J 14: 169–176
Secondary metabolism, reducing toxic compounds and enzymatic browning			
StISAC	increased and stabilized production of pigment production in cultured cells	Blue Star	D'Amelia et al. 2022 Plant Direct 6: e433
StPPO-1, StPPO-2	postharvest-related potential target gene	Yagana-INIA	Acha et al. 2022 Plants 10: 1882
StPPO2	reduced enzymatic browning	Desirée	González et al. 2020 Front Plant Sci 10: 1649.
St16DOX	gene discovery of α-solanine biosynthesis gene; α-solanine-free hairy root	Mayqueen	Nakayasu et al. 2018 Plant Physiol Biochem 131: 70–77
SSR2	lower level of predominant steroidal glycoalkaroids	Sassy	Yasumoto et al. 2020 Plant Biotechnol 37: 205–211
SSR2	lower level of predominant steroidal glycoalkaroids	Sassy	Yasumoto et al. 2019 Plant Biotechnol 36: 167–173
SSR2	lower level of predominant steroidal glycoalkaroids	Sassy	Sawai et al. 2014 Plant Cell 26: 3763–3774
Disease resistance			
StERF3	late blight disease resistance (*Phytophthora infestans*)	Lady Rosetta	Razzaq et al. 2022 Mol Biol Rep 49: 11675–11684
StHRC	multiple disease resistance	Russet Burbank	Kushalappa et al. 2022 Sci Rep 12: 20402
StSR4	late blight disease resistance (*Phytophthora infestans*)	Desirée	Moon et al. 2022 Front Plant Sci 13: 997888
eIF4E1	broader spectrum of potato virus Y resistance (group NTN strain)	Desirée	Lucioli et al. 2022 Front Microbiol 13:8 73930
StCCoAOMT	late blight resistance, enhanced by increased biosynthesis of cell wall phenylpropanoids	Russet Burbank	Hegde et al. 2021 Plant Cell Rep 40: 237–254
StNPR3	potato zebra chip disease resistance (Candidatus Liberibacter solanacearum)	Atlantic	Irigoyen et al. 2020 Nat Commun 11: 5802
StDMR6-1, StDND1, StCHL1	screening of candidate susceptibility genes to late blight disease	Desirée, King Edward	Kieu et al. 2021 Sci Rep 11: 4487
DMR6-1	broad spectrum of disease resistance	Saturna, Wotan	Carlsen et al. 2022 Front Genome Ed 3: 795644

DMR6-1	used as a model target gene to test new technology	Desirée	Veillet et al. 2020 Plant Cell Rep 38: 1065–1080
DMR6-1	used as a model target gene to test new technology	Desirée	Veillet et al. 2020 Int J Mol Sci 20: 402
Coilin	used as a model target gene to test new technology	Chicago	Khromov et al. 2018 Dokl Biochem Biophys 479: 90–94
Coilin	potato virus Y resistance, salt and osmotic stress tolerance	Chicago	Makhotenko et al. 2019 Dokl Biochem Biophys 484: 88–91
Herbicide target genes			
StALS	enabling chlorsulfuron screening to test prime editer	Desirée	Perroud et al. 2022 Plant Sci 316: 111162
StALS1, StALS2	enabling chlorsulfuron screening to test base editer	Desirée	Veillet et al. 2019 Int J Mol Sci 20: 402
ALS1, EPSPS1	chlorsulfuron and glyphosate targets, used as a model target gene	DMRH S5 28-5	Nadakuduti et al. 2019 Front Plant Sci 10: 110
StALS	used as a model target gene to test new technology	Desirée	Zong et al. 2018 Nat Biotechnol 36:950–953
ALS1	used as a model target gene to test new technology	Desirée	Butler et al. 2016 Front Plant Sci 7: 1045
StALS1	used as a model target gene to test new technology	Desirée, MSX914-10	Butler et al. 2015 PLoS One 10: e0144591
ALS	used as a model target gene to test new technology	Desirée	Nicolia et al. 2015 J Biotechnol 204: 17–24
Self-incompativility			
S-RNase	converting self-incompatible into self-compatible	DRH195, DRH310	Enciso-Rodriguez et al. 2019 Front Plant Sci 10: 376
S-RNase	converting self-incompatible into self-compatible	Phureja S15-65	Ye et al. 2018 Nat Plants 4: 651654
Sli	converting self-compatible into self-incompatible	B663, B665	Eggers et al. 2021 Nat Comm 12: 4141

CTTGCCTACTGTAATCGGTGATAAATGTGAATGCTTCCCCTTCTTCTTCTTCTCAGAAAT
CAATTTCTGTTTTGTTTTTGTTCATCTGTAGCTTGGTAGATTCCCCTTTTTGTAGACCAC
ACATCACATGGCAAGCATCACAGCTTCACACCACTTTGTGTCAAGAAGCCAAACTTCACT
AGACACCAAATCAACCTTGTCACCGATAACTCTTCCCGAGGCTCTGATACCAGTTTGTCG
GGAAAACACGGACAAACACAAAAATATATATGGTAAAAGTAATGGAAATAAAATGGGAAA
TAATGACACCAAGAATTTTACGTGGAAACCCTTCTAAATAAGGGAAAAACTACGGGCCAA
GAGGAGCAACTGATATTACTATAGTAAGGAATTTTACACTGTAGTCACGAGTACAATACT
CAAAATGACTACTACACACTCAAAAGGAATAACACTCTTTTGATTTCCACCTTACTAAAA
TATCGCTCACACTCTATTTTTCTTCACAGACTATTTTTCTTGTATAGCCTATGGAAACCT
CACAGCTCTCTAAATATTTTACTCTCTCTGTGAATTGGTGTATTTGGGAATGAGCAAAGG
GCTCTCTATTTATAGAAGAAATTTGCAGTTCTATACATAATATGTCAAAGTTGTGCCACC
GAAAGCATTTTTAATTGCTGTGTTTTTCAACAATTGCTGCTATTGCAATTTGCCAACAAT
TGACAATTGCATGCAGCAATTGTTGACACTAAAAAAAGAAAAGTGGGACTGGACCCCACA
AATCTCCCCCTCCAGTCCCATTTACTGGAAGGAGGTATCTTCATATTCTTTAGAGAGAGC
TCATACCCACAAGTTCTTTGCATAACTCGAACTTGTCTTTCAGTATCGTCTTGGTCAGCA
TATCTGCAGGATTTTCACTTGTGTGGATCTTTTTGACGTGAAATGATTCACTTTCCACTT
CCTCACGAATCCAATGATATCTGACGTCGATGTGTTTTGTCCTTGCATGGTACATGGAGT
TCTTGCTCAAGTCTATTGCACTCTGACTGTCACAATAGACAATATACTCCATCTGATGCA
AACCAAGCTCTTGAAGAAATCGCTTGAGCCATATCATCTCCTTGCCGGCTTCAGTAGCCG
CAATATACTCAGCTTCAGTTGTAGATAGTGCAACACACTTCTGCAACTTCGACTGCCATG
ATATAGCTCCCCCCTGAAAAAGTAAACAAATATCCAGTAGTGGATTTTCTGTTATCAAGG
TCACCTGCCATATCAGCATCTGTATAGCCCTTCAAGATTGGATCTGATGCTCCAAAAACA
CAAACATTCATCTGAGCTTCCTCTAAGATACCTGAGTATCCACTTCACAGCTTCCCAATG
CTCTTTTCCCGGATTGTCGAGAAATCTGCTAACAACACCAACTGCGTGAGCAATATCAGG
TCTAGTGCATACCATTGCATACATTAGACTTCCGACGACGGAGGAATATGGAACTTTGGC
CATGCTCTCTTTTTCCTCCCTAACTGTAGGACACATCTTTTTACTCAACTTCATATGACC
AGCAAGAGGTGTGCTAACGGGCTTAGCATTCTTCATATTGAAGCGCTCTAGTACACGTTC
AATGTACTTCTTCTGTGACAAATAAATCTTCCTTTCATCTCTGAGACGAGTAATTCTCAT
GCCTAAACCTTGCAGTAAGTCTTTCATTTGGTTACCTACTCATTCATTACTTATTTTGTT
TAGTTAGGTTCTACTGCATCAGTCTTTTTATCATTTAGGCCCGCGGACATCGGGTAATGA
CAATATCCCCCCGTTATGACCAATACAAAGATACTTGGGATACTAGCGTTGCGGTTGAGG
TACATCTTTCTATATTGATACGGTACAATATTGTTCTCTTACATTTCCTGATTCAAGAAT
GTGATCCGCTACTTTATCTGCAGGTCAAAGTTGGAGACAGCATTGAAATTGTTCGTTTCT
TTCACTGCTATAAACGTGGGGTTGATCGTGTTTTTGTTGACCACCCAATGTTCTTGGAGA
AAGTAAGTAAGTATATTATGATTATGAATCCATCCTGAGGGATACGCAGAACAGGTCATT
TTGAATATCTTTTAACTCTACTGGTGCTTTTACTCTTTTAAGGTTTGGGGTAAAACTGGT
TCAAAAATCTATGGCCCCAAAGCTGGACTAGATTATCTGGACAATGAACTTAGGTTCAGC

Supplementary Figure S1. Nucleotide sequence of the rearrangement region in the allele #105d of mutant line #105. The nucleotide sequence of the insertion in the *GBSS1* gene is indicated by blue letters. This mutant allele contains a 250-nt deletion along with a 1,484-nt insertion which starts from the position -56 in the target region. The inserted sequence shows similarity to an unidentified sequence of *Solanum tuberosum* genome.

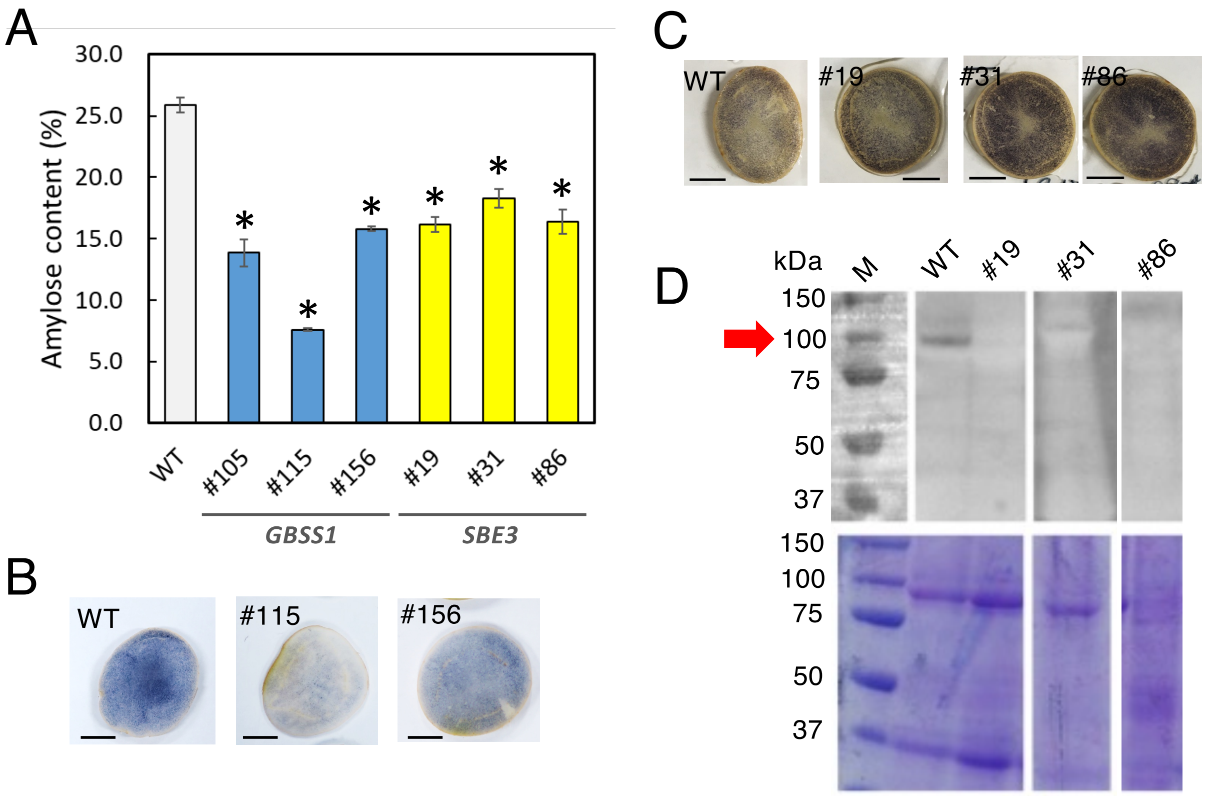

Supplementary Figure S2. Traits of the *GBSS1* and *SBE3* mutants. (A) Amylose content of starch in tubers of *GBSS1* and *SBE3* mutants. Error bars indicate standard deviation (n=3). Asterisks indicate significant differences in the values of transformants compared to that of wild type at $P < 0.05$. (B) Iodine staining of tubers of *GBSS1* mutants. Bar = 2 cm. (C) Iodine staining of tubers of *SBE3* mutants. Bar = 2 cm. (D) Western blot analysis of the proteins in tubers of *SBE3* mutants. A rabbit antiserum against the rice BEI was used for the detection of SBE3. The arrow corresponds to SBE3. The lower panel shows the corresponding SDS-PAGE results. M: molecular weight marker. WT: wild type. #105, #115 and #156 indicate the *GBSS* mutant lines, and #19, #31 and #86 are the *SBE3* mutant lines. Detailed procedures used in this figure is described in Supplementary Methods.
